# Does Liver Transplantation in the Rat Cause a Regenerative Response

**DOI:** 10.1155/1994/96762

**Published:** 1994

**Authors:** G. H. C. Engelbrecht, Heather Mcleod, Marilyn Tyler, Zoe Lotz, K. Jaskiewicz, Rosemary Hickman

**Affiliations:** 1 Departments of Surgery and Medical Research Council Liver Centre University of Cape Town South Africa; 2 Department of Anatomical Pathology Council Liver Centre University of Cape Town South Africa; 3 Department of Surgery University of Cape Town Medical School Observatory Cape Town 7925 South Africa

## Abstract

This study was conducted to determine the pattern of early regenerative response to orthotopic intact liver transplantation in the rat and to investigate whether the response differed in grafts with or without revascularisation of the arterial bed.

Outbred male Long Evans (LE-LE allogeneic, non rejector) rats weighing 300–350g were subjected to orthotopic intact liver allograft using a “sleeve” anastomosis for the hepatic artery. Total warm ischaemia ranged from 19 to 34 minutes and no storage was employed. Comparison was made with a group of control rats which were subjected to 25 minutes total inflow occlusion and regeneration was measured with tissue thymidine kinase (TK) and mitotic figures. Samples were taken at 1,2,4,7,10 and 20 days post-operatively. Plasma aspartate aminotransferase (AAT) and light microscopy were used to evaluate hepatocyte necrosis. There was a brief sharp increase in TK and AAT in the first 24 hours after sham operation but no appearance of mitotic figures. A similar but more prolonged increase in TK occurred in the arterialised transplant group with the highest levels recorded on day 4. The level remained significantly elevated above pre-operative until 10 days and declined within 20 days. Mitotic figures appeared at 2 days, reached significance at 7 and 10 days and had disappeared by 20 days. The pattern of changes was accentuated in animals in which the artery was not reanastomosed and the increases in TK and AAT were still significant at 20 days. Whilst similar degrees of peri-portal cellular infiltrate occurred in both groups of rats, bile duct proliferation was most obvious in non-arterialised animals.

As compared with a previously prepared group of partially hepatectomised animals, the regenerative response after liver transplant was delayed and prolonged especially in the non-arterialised group.

It is concluded that a regenerative response occurred in liver allografts in rats soon after operation, which was slightly prolonged if the hepatic artery was not anastomosed and that the response seemed to be related to hepatocyte damage which occurred as part of the procedure. The relevance of these findings to clinical liver transplantation is discussed.


*HPB Surgery*, 1994, Vol. 7, pp. 211–217
Reprints available directly from the publisher
Photocopying permitted by license only
© 1994 Harwood Academic Publishers GmbH
Printed in the United States of America

### DOES LIVER TRANSPLANTATION IN THE RAT CAUSE A REGENERATIVE RESPONSE

### The Effect of Arterialisation of the Graft

G.H.C. ENGELBRECHT, HEATHER MCLEOD, MARILYN TYLER, ZOE LOTZ, K. JASKIEWICZ* and ROSEMARY HICKMAN

*Departments of Surgery and Anatomical Pathology* and Medical Research Council Liver Centre, University of Cape Town, South Africa*

*(Received 21 April 1993)*

This study was conducted to determine the pattern of early regenerative response to orthotopic intact liver transplantation in the rat and to investigate whether the response differed in grafts with or without revascularisation of the arterial bed.

Outbred male Long Evans (LE-LE allogeneic, non rejector) rats weighing 300–350g were subjected to orthotopic intact liver allograft using a "sleeve" anastomosis for the hepatic artery. Total warm ischaemia ranged from 19 to 34 minutes and no storage was employed. Comparison was made with a group of control rats which were subjected to 25 minutes total inflow occlusion and regeneration was measured with tissue thymidine kinase (TK) and mitotic figures. Samples were taken at 1,2,4,7,10 and 20 days post-operatively. Plasma aspartate aminotransferase (AAT) and light microscopy were used to evaluate hepatocyte necrosis. There was a brief sharp increase in TK and AAT in the first 24 hours after sham operation but no appearance of mitotic figures. A similar but more prolonged increase in TK occurred in the arterialised transplant group with the highest levels recorded on day 4. The level remained significantly elevated above pre-operative until 10 days and declined within 20 days. Mitotic figures appeared at 2 days, reached significance at 7 and 10 days and had disappeared by 20 days. The pattern of changes was accentuated in animals in which the artery was not reanastomosed and the increases in TK and AAT were still significant at 20 days. Whilst similar degrees of peri-portal cellular infiltrate occurred in both groups of rats, bile duct proliferation was most obvious in non-arterialised animals.

As compared with a previously prepared group of partially hepatectomised animals, the regenerative response after liver transplant was delayed and prolonged especially in the non-arterialised group.

It is concluded that a regenerative response occurred in liver allografts in rats soon after operation, which was slightly prolonged if the hepatic artery was not anastomosed and that the response seemed to be related to hepatocyte damage which occurred as part of the procedure. The relevance of these findings to clinical liver transplantation is discussed.

KEY WORDS: Liver transplant, rat, liver regeneration, arterialisation

### INTRODUCTION

A regenerative response has been shown to occur in pigs with intact liver grafts[1] and has been alluded to in rats with auxiliary grafts[2] or intact orthotopic grafts[3]. Since the original description of the technique of liver transplantation in the rat by Sun Lee[2] and the extensive experience reviewed by Kamada *et al.*[4], the model has

Address correspondence to: Professor R. Hickman, Department of Surgery, University of Cape Town Medical School, Observatory, 7925, Cape Town, South Africa.

become widely used. There is however, increasing evidence of the benefits of revascularisation of the arterial bed[5,6]. Despite Kamada's survival rate of 95.3%, he and others[7,8,9] have reported a significant incidence of biliary complications in this model probably related to the absence of arterial supply to the donor bile duct. More recently Gassel[10] and Sumimoto *et al.*[11], have noted differences in expression of Class I and Class II MHC antigens in animals with or without re-arterialisation of the transplanted liver.

The present study was conducted to define the time course of the regenerative response after intact liver grafting in rats and to determine any differences between arterialised and non-arterialised grafts.

### MATERIALS AND METHODS

### *(a) Liver Graft*

Male Long Evans (LE to LE allogeneic, non rejector) rats weighing (300–350g) were used after an overnight fast, as follows:-

*Group I Liver transplant (n = 110 recipients)*
Animals were subjected to liver transplant under ether anaesthesia using the method previously described for autografts[12]. Briefly, the donor liver was dissected free and the recipient was prepared simultaneously. After i.v. heparinisation (0.5 mg/kg) of both animals, the donor liver was removed without flushing; it was held in cooled swabs during the immediate insertion. The portal vein and suprahepatic i.v.c. were anastomosed with 9–0 silk and the circulation to the liver was restored. The infrahepatic i.v.c. was anastomosed with 9–0 silk and opened. In 55 animals, the hepatic artery was repaired using the "sleeve" technique of Duminy[13]. Thereafter in all animals, the bile duct was repaired by end-to-end anastomosis over a stent[12]. The laparotomy was closed.

*Group II Control - sham-operated (n = 100 rats)*
The liver dissection and heparinisation were performed as if for transplantation and the portal vein and hepatic artery were clamped for 25 minutes to simulate the period of warm ischaemia during grafting. Thereafter, the portal vein clamps were removed in all animals and the hepatic arterial clamps in 50 rats. In the remaining 50, the hepatic artery was definitively ligated and divided. The laparotomy was closed.

All operations were performed between 9.00 and 11.00 daily and post-operatively, animals were housed in a room with 12 hour day/night lighting schedule.

### *(b) Sampling*

Forty animals were sacrificed under ether anaesthesia on post-operative days 1,2,4,7 and 10. At each time point, 20 liver transplant recipients (10 arterialised (AG), 10 non-arterialised (NAG) and 20 controls (10 with the artery intact (AS) and 10 with the artery divided (NAS) were used. The final group of 10 liver transplant recipients (5 AG, 5 NAG) was sacrificed at 20 days post-operatively. At sacrifice, where relevant, the patency of the hepatic artery or the arterial anastomosis was confirmed and the graft site inspected for evidence of biliary leakage. Blood was taken by cardiac puncture for measurement of aspartate aminotransferase (AAT) and liver biopsies were taken from the left lobe, frozen in liquid nitrogen for measurement of thymidine kinase[14] and into formalin for histology and for counting of mitotic figures.

The changes in thymidine kinase were compared with data obtained in several previous studies of liver regeneration in the rat in which the early response to 66% partial hepatectomy was defined by measurement of DNA synthesis rate, and thymidine kinase levels and mitotic indices in liver biopsies[14]. In these studies it was shown that measurement of thymidine kinase accurately represented the levels of DNA synthesis obtained after injection of $C^{14}$thymidine. Thus tissue thymidine kinase has been used routinely in this laboratory for assessment of the regenerative response. The mean value for thymidine kinase in normal LE rats in this laboratory is 0.242 0.032 dpm.mg protein $\times 10^5$, for mitotic index is $< 1$ and for aspartate aminotransferase which was used as an indicator of hepatocyte necrosis, is $50 \pm 6$ units.

Comparison of results was made using analysis of variance and the Mann Whitney test and $p < 0.05$ was taken as significant.

### RESULTS

The mean ischaemic time from clamping of the donor portal vein to its release in the recipient was 21 minutes (range 15–27 minutes); anastomosis of the hepatic artery lasted a mean of 5 minutes and 36 seconds (range 4–7 minutes). All animals survived the operation and until sacrifice. At the time of sacrifice, all animals were eating and drinking normally and were active in the cages. All anastomoses were patent and pulsation was clearly visible in the hepatic arteries.

The recorded peak in thymidine kinase activity after PH occurred at 24 hours ($2.430 + 0.320$ dpm/mg $\times 10^5$). Thereafter a decline commenced which became significantly different at 4 days from the peak.

In recipients of arterialised grafts (Figure 1), the mean level of TK at 24 hours of $0.321 + 0.050$ dpm/mg $\times 10^5$ ($p < 0.05$ compared with levels in partially hepatectomised rats) increased sharply to $1.250 + 0.09$ dpm/mg $\times 10^5$ at 2 days ($p < 0.05$ compared to normal) and to recorded peak of $1.815 + 0.12$ dpm/mg $\times 10^5$ ($p < 0.01$ compared to normal) at 4 days. Levels were still significantly increased above normal at 10 days ($1.005 + 0.102$ dpm/mg $\times 10^5$); at 20 days, TK levels in AG recipients were still elevated ($0.649 + 0.138$ dpmmg $\times 10^5$) but not significantly so. The mitotic index was $5 + 2$ at 2 days and remained significantly elevated ($p < 0.05$) within this range until 10 days, becoming insignificantly increased at 20 days. Plasma AAT showed a significant 10-fold increase to $535 + 47$ units/ml within 24 hours and declined slowly thereafter. By 10 days, the levels were still 2-fold increased ($155 + 40$ units/ml) but by 20 days was $84 + 13$ units/ml and within normal range of $50 + 6$ units/ml.

In recipients of non-arterialised grafts TK levels increased more slowly — while the value at 2 days ($0.173 + 0.224$ dpm/mg $\times 10^5$) was significantly greater than normal levels, it was not greater than TK levels in AG recipients at that time; the

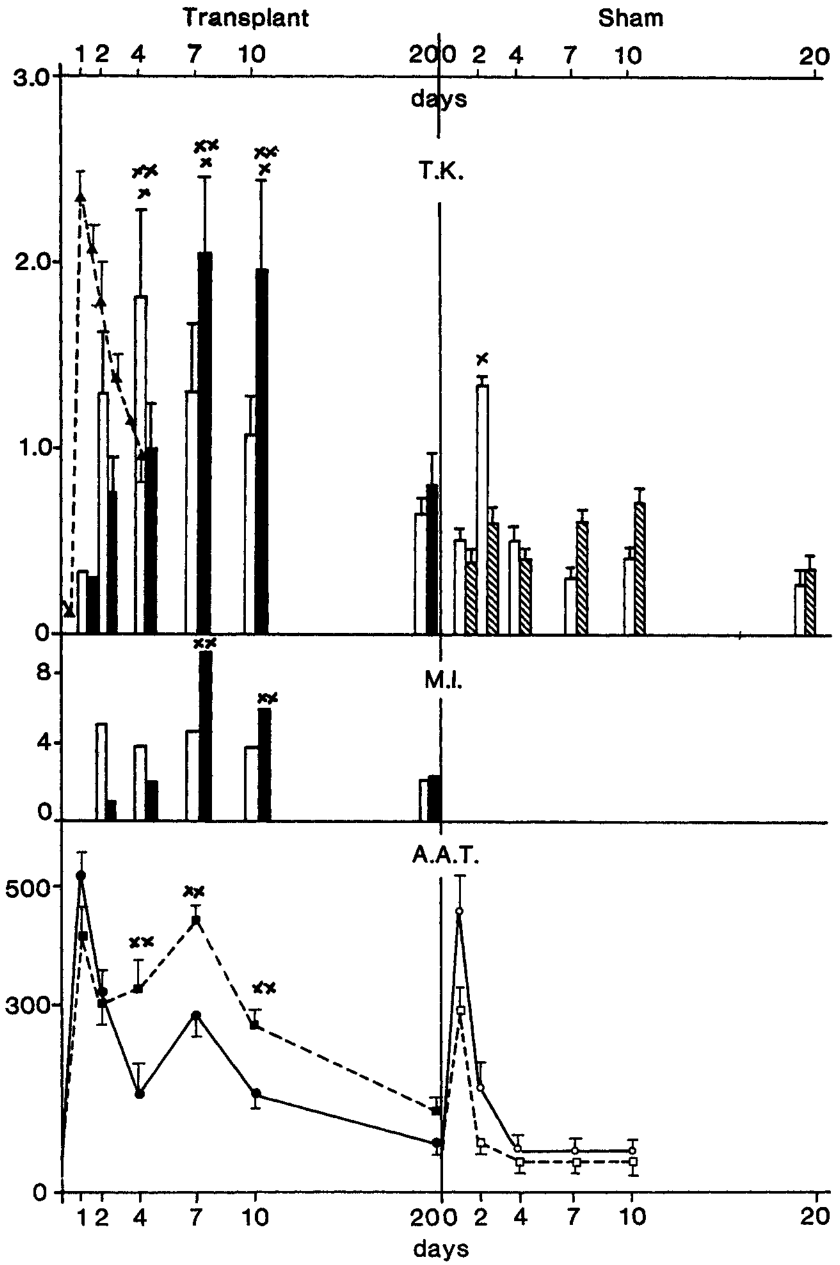

**Figure 1** In all graphs *$p < 0.05$ compared with pre-operative values **$p < 0.05$ arterialised versus non-arterialised. (a) The changes in tissue thymidine kinase (dpm/mg protein × 105) in recipients of arterialised □ or non-arterialised ■ grafts and in control rats with the artery patent □ or ligated ▧; ▲—▲ = partial hepatectomy. Values are given as mean ± s.e.m. (b) The mitotic indices (figures /1000 cells) in recipients of arterialised □ or non-arterialised ■ grafts. values are given as mean; s.e.m. is omitted for clarity. (c) The mean ± s.e.m. plasma levels of aspartate aminotransferase (units/ml) in control animals (artery patent O—O) or (artery ligated □—□). or grafted recipients with the artery patent (O—O) or ligated (■--■). All values for AAT were significantly elevated above normal (* omitted for clarity).

highest recorded values occurred at 7 and 10 days. These values were significantly higher than the level at 2 days (in the NAG group), but not different from the TK levels at 4,7 or 10 days in the AG group. Levels were persistently elevated at 20 days but were not different from those in the AG group. The pattern of changes in MI differed slightly from that in the AG group in that the peak increase (9 + 3) was only recorded at 7 days, being significantly higher than values at 2 and 4 days in the NAG group and all values in the AG group. The increase in AAT in the non-arterialised recipients at 24 hours was similar to that in the AG group but remained at a high plateau until 7 days and the decline only commenced at 10 days. Even at this time, levels were still significantly higher than those in the AG group but by 20 days, levels were similar to those in the AG group but still significantly higher than normal.

In the control group, although some TK activities were elevated, only two were significantly different from normal. There was a single significant peak of 1.385 + 0.26 dpm/mg $\times 10^5$ at 2 days in the AS group and one of 0.734 + 0.152 dpm/mg $\times 10^5$ at 10 days in the NAS group. Less than 2 mitotic figures/1000 cells were found in all biopsies from control animals. The peak increases and pattern of AAT values in the AS group were not different from those in the AG group. In the NAS group, values returned to normal within 4 days.

### HISTOLOGY

All animals showed evidence of marginal or centrilobular necrosis in liver biopsies at 24 hours and 2 days. In control animals, these changes resolved rapidly and biopsies at 4,7 and 10 days showed minimal differences from normal. In liver transplanted animals however, *whether arterialised or not*, there was evidence of hepatocyte necrosis with peri-portal mononuclear cell infiltrate. By 10 days, these changes affected approximately 50% of hepatocytes and portal tracts and were graded as moderate. The changes were not more severe by 20 days. Bile duct proliferation was seen in only one biopsy from the AG group, but was a uniform feature of the NAG group as was inflammatory cell infiltrate into the sinusoidal areas.

### DISCUSSION

This study was conducted to define the regenerative response to intact liver grafting in rats and to determine whether restoration of the arterial circulation influenced this response.

The data showed that there was a significant regenerative response in recipients of liver grafts, whether arterialised or not. While the response in the NAG group appeared delayed compared with that in the AG group, the differences were not significant due to wide ranges. Of more importance however, were the facts that in both groups, the peak response was recorded at least 24 hours after the recorded peak in PH animals and that the responses persisted at least to 10 and even to 20 days when the response after PH was resolving by 4 days. It is likely that this persistent response was related to the hepatocyte necrosis (shown by elevation of aspartate aminotransferase and on microscopy) which continued up to 20 days.

It was decided to conduct these grafts without the complicating factors of flushing and storage of the liver but this decision necessitated a period of warm ischaemia of up to 27 minutes. In the control group, there was evidence of an increase in TK at 2 days in the AS group which was similar to the increase in the grafted groups but which was significantly higher than the peak seen in the NAS group. Neither of these two increases in TK in the control group was accompanied by an increase in mitotic figures. They cannot therefore be interpreted as a regenerative response. Other studies in our laboratory have shown that ischaemia of 20 minutes does not affect the regeneration of PH livers and that the duration must be prolonged to 40 minutes before an effect is seen[15]. Thus ischaemia alone did not seem to be responsible for the observed changes.

So, to return to these changes. It appears that grafting of the liver in this manner results in a regenerative response of similar magnitude to that noted after PH under similar circumstances[14] but that the commencement of the response is delayed and it is prolonged. In their description of DNA synthesis in rat liver allografts between strain combinations showing variable degrees of rejection, Teramoto *et al.*[3] concluded that regeneration and rejection occurred in parallel from 7 days post-operatively. The present study highlights that the regenerative response commenced much earlier and persisted at least to 10 days; there was no clear evidence of rejection on histology.

What is the relevance of arterialisation to the regenerative response? The present data did not demonstrate a significant difference between recipients of arterialised or non-arterialised grafts in regard to TK or MI. With similar round cell infiltrates noted on histology, the only histological differences between the two groups of grafts were that proliferation of bile ducts and Kupffer cells only occurred in biopsies from non-arterialised grafts. These features confirm previous observations that the most commonly reported complications in non-arterialised grafts are necrosis and obstruction of bile ducts[7,8,9] and that these are prevented by arterial anastomosis[7,9]. Engemann *et al.*[16] and Lie *et al.*[17] have reported improved survival in animals with arterialised grafts and Gassel *et al.*[10], Sumimoto *et al.*[11] and Engemann *et al.*[18] have reported the appearance of class II MHC antigens and a periportal round cell infiltrate, class I MHC antigens or autoreactive T cells in non-arterialised grafts. The present studies were not designed to confirm the survival data but the appearance of the mild peri-portal round cell infiltrate may have been in response to antigens as described above.

It is concluded that a regenerative response does occur in liver allografts in rats soon after operation; this response is slightly prolonged if the hepatic artery is not reanastomosed. It seems important to identify the histological changes (in non-arterialised grafts) which result from interruption of the arterial supply. Hepatocyte necrosis and peri-portal inflammatory cell infiltration occurred as a result of damage during grafting in both groups but proliferation of bile ducts and Kupffer cells seemed specific for de-arterialisation. Thus the initial period of regenerative response is not affected by de-arterialisation but both groups of grafts showed a delay in regenerative response which would persist in the clinical setting into the time when rejection would be expected. Koch and Leffert[19] have recently questioned whether liver regeneration is in fact beneficial to the transplanted liver and other studies have shown disturbed liver function during the regenerative response. On the other hand, some authors[20,21] suggest that liver regeneration may cause immunological suppression which might be beneficial. Further studies are needed to determine whether a regenerative response is indeed advantageous after liver transplantation.

### *Acknowledgements*

Messrs Ryneveldt, Hendricks and Middlekoop provided operative assistance and post-operative care of the animals. Funding was also received from the Staff Research Fund of the University of Cape Town and from the Mauerberger Foundation Fund. Mrs P. Johnson prepared the graph and Mrs G. Mellett and Mrs V. Johns typed the manuscript.

### *References*

1. Kahn, D., Hickman, R., McLeod, H. and Terblanche, J. (1982) The stimulatory effect of a partially hepatectomised auxiliary graft upon the host liver; observations on the regenerative response in orthotopic grafts. *S. Afr. Med. J.*, **61**, 362–365
2. Lee, S., Edgington, T.S. and Orloff, M.J. (1968) The role of afferent blood supply in regeneration of liver isografts in rats. *Surg. Forum*, **19**, 360–363
3. Teramoto, K., Shimizu, K., Tsukada, K. and Kamada, N. (1990) DNA synthesis in hepatocytes during liver allograft rejection in rats. *Transplantation*, **50**, 199–201
4. Kamada, N. and Calne, R.Y. (1983) A surgical experience of 530 liver transplants in the rat. *Surgery*, **9**, 64–69
5. Wheatley, A. Personal communication
6. Liu, T.S., Freise, C.E., Ferrell, L., Ascher, N. and Roberts, J.P. (1992) A modified vascular "sleeve" anastomosis for rearterialisation in orthotopic liver transplantation in rats. *Transplantation*, **54**, 179–180
7. Steffen, R., Ferguson, D.M. and Krom, R.A.F. (1989) A new method for orthotopic rat liver transplantation with arterial cuff anastomosis of the recipient common hepatic artery. *Transplantation*, **45**, 166–168
8. Hasuike, Y., Monden, M., Valdivia, L.A., Kubota, N., Gotoh, M. and Nakano, Y. (1988) A simple method for orthotopic liver transplantation with arterial reconstruction in rats. *Transplantation*, **45**, 831–836
9. Howden, B., Jablonski, P., Grossman, H. and Marshall, V.C. (1989) The importance of the hepatic artery in rat liver transplantation. *Transplantation*, **47**, 428–434
10. Gassel, H.J., Engemann, R. and Thiede, B. (1987) MHC class II antigen expression on Kupffer cells after orthotopic rat liver transplantation: a consequence of non-specific inflammation or allograft rejection. *Transplant Proc.*, **19**, 3017–3018
11. Sumimoto, R., Shinomiya, T. and Yamaguchi, A. (1991) Influence of hepatic arterial blood flow in rats with liver transplants. *Transplantation*, **51**, 1138–1139
12. Engelbrecht, G.H.C., Duminy, F., Hickman, R. and Terblanche, J. (1989) A technique for liver autograft in the rat including a novel method for bile duct anastomosis. *S. Afr. J. Sci.*, **85**, 391–395
13. Duminy, F.J. (1989) A microvascular "sleeve" anastomosis. *J. Surg. Res.*, **46**, 189–194
14. Kahn, D., Stadler, J., Terblanche, J. and Hickman, R. (1980) Thymidine kinase, an inexpensive index of liver regeneration in the large animal model. *Gastroenterology*, **79**, 907–913
15. Bolitho, D.G., Engelbrecht, G.H.C., Lotz, Z., Tyler, M., Hickman, R. and Terblanche, J. Does hepatic ischaemia influence hepatic regeneration in rats? *Hepatology*, (In Press, 1993)
16. Engemann, R., Ulrichs, K., Thiede, A., Muller-Ruchholtz, W. and Hamelmann, H. (1982) Value of a physiological liver transplant model in rats. *Transplantation*, **32**, 566–568
17. Lie, T.S., Hansen, H.H. and Niehaus, K.J. (1983) Bedeutung der arterialisation des transplantation bei Rattenlebertransplantation. Langenbecks *Archiv. fur Chirurgie*, **359**, 133–142
18. Engemann, R., Ulrichs, K., Thiede, A., Muller-Ruchholtz, W. and Hamelmann, H. (1983) A mechanism of tolerance in arterialised rat liver transplantation. *Transpl. Proc.* **XV**, 729
19. Koch, K.S. and Leffert, H.L. (1989) Do human liver transplants regenerate? *Hepatology*, **9**, 789–791
20. Davies, H.F.F.S., Pollard, S.G. and Calne, R.Y. (1989) Soluble H-LA antigens in the circulation of liver graft recipients. *Transplantation*, **47**, 524–527
21. Yokomura, K., Miyahara, S., Takahashi, H. and Kimura, Y. (1983) Regeneration and the immune system (ii) *Europ. J. Immunology*, **13**, 883–889

